# Identification and physiological characterization of phosphatidic acid phosphatase enzymes involved in triacylglycerol biosynthesis in *Streptomyces coelicolor*

**DOI:** 10.1186/1475-2859-12-9

**Published:** 2013-01-29

**Authors:** Santiago Comba, Simón Menendez-Bravo, Ana Arabolaza, Hugo Gramajo

**Affiliations:** 1 Microbiology Division, IBR (Instituto de Biología Molecular y Celular de Rosario), Consejo Nacional de Investigaciones Científicas y Técnicas. Facultad de Ciencias Bioquímicas y Farmacéuticas, Universidad Nacional de Rosario, Suipacha 531, S2002LRK, Rosario, Argentina

**Keywords:** PAP, Triacylglycerol, Oleaginous bacteria, Lipid metabolism

## Abstract

**Background:**

Phosphatidic acid phosphatase (PAP, EC 3.1.3.4) catalyzes the dephosphorylation of phosphatidate yielding diacylglycerol (DAG), the lipid precursor for triacylglycerol (TAG) biosynthesis. Despite the importance of PAP activity in TAG producing bacteria, studies to establish its role in lipid metabolism have been so far restricted only to eukaryotes. Considering the increasing interest of bacterial TAG as a potential source of raw material for biofuel production, we have focused our studies on the identification and physiological characterization of the putative PAP present in the TAG producing bacterium *Streptomyces coelicolor*.

**Results:**

We have identified two *S. coelicolor* genes, named *lppα* (SCO1102) and *lppβ* (SCO1753), encoding for functional PAP proteins. Both enzymes mediate, at least in part, the formation of DAG for neutral lipid biosynthesis. Heterologous expression of *lppα* and *lppβ* genes in *E. coli* resulted in enhanced PAP activity in the membrane fractions of the recombinant strains and concomitantly in higher levels of DAG. In addition, the expression of these genes in yeast complemented the temperature-sensitive growth phenotype of the PAP deficient strain GHY58 (*dpp1lpp1pah1*). In *S. coelicolor*, disruption of either *lppα* or *lppβ* had no effect on TAG accumulation; however, the simultaneous mutation of both genes provoked a drastic reduction in *de novo* TAG biosynthesis as well as in total TAG content. Consistently, overexpression of Lppα and Lppβ in the wild type strain of *S. coelicolor* led to a significant increase in TAG production.

**Conclusions:**

The present study describes the identification of PAP enzymes in bacteria and provides further insights on the genetic basis for prokaryotic oiliness. Furthermore, this finding completes the whole set of enzymes required for *de novo* TAG biosynthesis pathway in *S. coelicolor*. Remarkably, the overexpression of these PAPs in *Streptomyces* bacteria contributes to a higher productivity of this single cell oil. Altogether, these results provide new elements and tools for future cell engineering for next-generation biofuels production.

## Background

**Figure 1:**
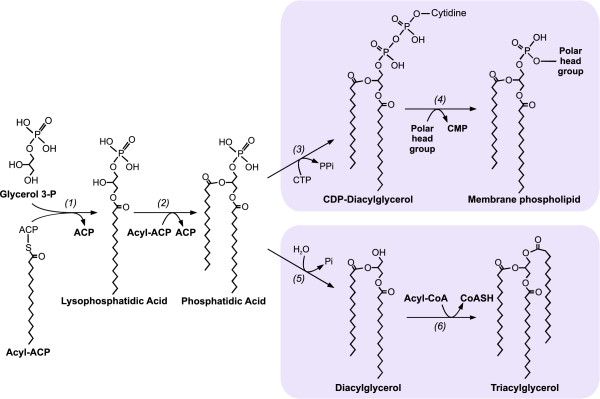
**Biosynthesis of membrane glycerophospholipids and triacylglycerols in *S. coelicolor*.** PA is the metabolic branch point dividing glycerophospholipid and triacylglycerol biosynthesis. *(1)* G3P:acyltransferase. *(2)* Lisophosphatidic acid acyltransferase*. (3)* Phosphatidate cytidylyltransferase*. (4)* Phosphatidyltransferase. *(5)* Phosphatic acid phosphatase. *(6)* Diacylglycerol:acyltransferase.

Triacylglycerols (TAG) are the most common lipid-based energy reserves in animals, plants, and eukaryotic microorganisms [1]. In bacteria, only a few examples of substantial TAG accumulation have been reported, mainly in members of the actinomycetes group of bacteria, such us *Mycobacterium*[2], *Nocardia*[3], *Rhodococcus*[4], and *Streptomyces*[5]. TAG biosynthesis occurs in nature by three different enzymatic activities: diacylglycerol:acyltransferase, phospholipid:diacylglycerol acyltransferase, and diacylglycerol:diacylglycerol acyltransferase [6-10]. These three reactions involve acylation of diacylglycerol (DAG), making this lipid a fundamental precursor of TAG biosynthesis. DAG moiety is produced by reactions that are partially shared with the glycerophospholipid biosynthesis pathway [11,12] (Figure 1), consisting in consecutive acylations of glycerol-3-phosphate (G3P), catalyzed by G3P:acyltransferase and lysophosphatidic acid (LPA) acyltransferase, generating phosphatidic acid (PA). PA can be dephosphorylated by the enzyme phosphatidic acid phosphatase (PAP) yielding DAG. Alternatively, PA is used for the synthesis of the liponucleotide intermediate CDP-DAG by the phosphatidate cytidylyltransferase enzyme [12]. Bacteria produce their membrane glycerophospholipids exclusively through CDP-DAG route, making PA the metabolic branch point dividing TAG and glycerophospholipid synthesis [13,14]. Thus, DAG formation is the first specific reaction of TAG biosynthesis in oleaginous bacteria, suggesting a key role of PAP activity in the regulation of PA flux towards TAG or glycerophospholipid synthesis [14] (Figure 1).

PAP enzymes (EC 3.1.3.4) have been identified and characterized in various eukaryotes. In yeast cells, PAP activity plays a central role in both lipid metabolism and intracellular signaling mechanisms [15,16]. Two distinct family of PAPs, referred to as PAP1 and PAP2, have been described [17-19]. The enzymes belonging to the PAP1 family show a Mg^2+^-dependent PAP activity, utilize PA as a unique substrate and localize in the soluble fraction of the cell [20-22]. In contrast, the PAP2 enzymes utilize an array of different substrates such as PA, LPA, sphingosine-1- phosphate and diacylglycerol pyrophosphate (DGPP) among others. This family, currently known as lipid phosphate phosphatases LPPs, do not require Mg^2+^ for activity and are integral membrane proteins [23].

The first member of the PAP1 family of enzymes (Pah1) has been purified and characterized from the membrane and cytosolic fractions of yeast [24]. The analysis of mutants lacking the *pah1* gene has provided evidences that this enzyme generates the DAG used for lipid synthesis [25]. Cells containing a *pah1* mutation accumulate PA and have reduced amounts of DAG and its acylated derivative TAG [25]. The genes encoding for the PAP1 family of enzymes are highly conserved among eukaryotic species, but they do not possess any homologues in bacterial genomes.

On the other hand, the main PAP2 enzymes are encoded in yeast by the *dpp1*[26] and *lpp1*[27] genes, with the former being the principal contributor of this activity [27]. The *dpp1* and *lpp1* gene products are integral membrane proteins with six transmembrane spanning regions and are localized in the vacuole [28,29] and Golgi [30] compartments of the cell, respectively. The Dpp1 enzyme shows a preference for DGPP as a substrate [31], whereas the Lpp1 enzyme has similar substrate specificity for both PA and DGPP [32]. Overall, these enzymes belong to the PAP2 superfamily (pfam 01569), which includes more than 600 eukaryotic and prokaryotic proteins. Within the members of this group, PgpB of *E. coli* is the only enzyme known to display PAP activity [33]. Originally, *pgpB* was identified in a screen designed to isolate cells defective in phosphatidylglycerol phosphate (PGP) phosphatase activity [34]. However, further analyses suggested that PgpB had a broad substrate spectrum, as demonstrated by its *in vitro* phosphatase activities towards PGP, PA, LPA, DGPP and undecaprenyl pyrophosphate (C55-PP) [33-36]. Touzé *et al.* reported that PgpB prefers pyrophosphate lipids as substrates and indicated that this enzyme is involved in the C55-P metabolism [35].

Interestingly within this superfamily, Nakamura *et al.*, identified and characterized plastidic PAP2 enzymes in *Arabidopsis thaliana* (LPPsβ, γ, δ, ε1 and ε2) and its homologue in *Synechocystis* sp. PCC6803 (synLPP) [37]. The author suggested that theses enzymes belong to a prokaryotic subfamily of PAP2 and could be involved in providing DAG precursors for monogalactosyl and digalactosyl diacylglycerol synthesis [37].

Remarkably, despite the functional relevance of these proteins in lipid metabolism of oleaginous bacteria, no studies were conducted towards the identification and characterization of PAPs in this group of bacteria. In an effort to unravel the biochemical properties and physiological significance of these proteins in *S. coelicolor*, we carried out a comprehensive bioinformatic analyses to identify and further characterize the PAP enzyme(s) of this microorganism. In this study we present the genetic and biochemical characterization of two *Streptomyces* PAPs, specifically demonstrating that SCO1102 (*lppα*) and SCO1753 (*lppβ*) encode PAP enzymes catalyzing the formation of DAG from PA and that both proteins are essential for achieving wild type levels of TAG in this microorganism.

## Results and discussion

### Identification of putative PAPs in *S. coelicolor*

**Figure 2:**
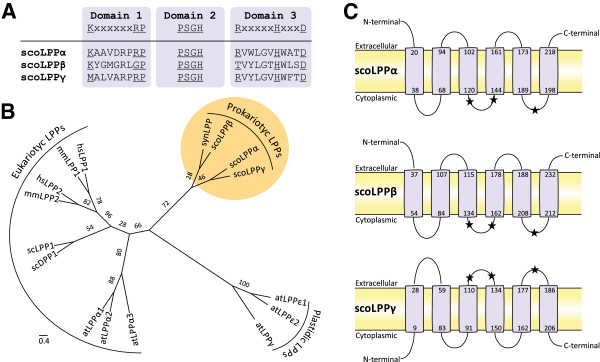
**Bioinformatic analysis of *S. coelicolor *PAPs.** (**A**) Sequence alignment of scoLPPs PAP2 domains. The key residues for this catalytic activity are underlined. (**B**) Phylogenetic tree of LPP enzymes from different organisms. Bootstrap values are shown along the branches. (**C**) Transmembrane topology prediction of scoLPPs. Numbers represent amino acid position of start and end of the respective transmembrane helix. Location of the catalytic domains is represented with black stars. Sequence accession numbers: atLPP_alfa1 [EMBL:Q9ZU49], atLPP_alfa2 [EMBL:Q9XI60], atLPP_alfa3 [EMBL:Q8LFD1], atLPP_epsilon1 [EMBL:F4J220], atLPP_epsilon2 [EMBL:Q6NQL6], atLPP_gamma [EMBL:Q6NLA5], scLPP1p [EMBL:Q04396], scDPP1p [EMBL:Q05521], hsLPP1 [EMBL:O14494], hsLPP2 [EMBL:O43688], mmLPP1 [EMBL:Q61469], mmLPP2 [EMBL:Q9DAX2], scoLPPα [EMBL:Q9K3P6], scoLPPβ [EMBL:Q9EWX3], scoLPPγ [EMBL:O86624], synLPP [EMBL:Q55398].

Analysis of the *S. coelicolor* genome with Pfam [38] and Conserved Domain Database [39] tools reveals that it encodes at least 14 proteins with putative PAP2 domains (pfam1569). As we already mentioned, this class of enzymes are involved in a myriad of reactions with different lipid phosphates as substrates. Thus, in order to identify the putative PAP enzymes responsible for DAG production, we performed an homology sequence search over the *S. coelicolor* genome database with the amino acid sequence of the synLPP protein as query, using BLAST [40] and FASTA [41] algorithms. FASTA analysis presented significant matches (E-value < 10^-3^) to Sco1102, Sco1753 and Sco6355 proteins, and the BLAST tool output also indicated these three proteins as the best hits. The three candidates were named Lppα (Sco1102), Lppβ (Sco1753) and Lppγ (Sco6355) and have a predicted molecular weight of 24,8,37,8 and 28,3 kDa, respectively. Detailed analysis of the protein sequences indicates that they conserve the key amino acids of the PAP2 catalytic domain and are all predicted to be integral membrane proteins [42] (Figure 2).

Phylogenetic analysis using curated sequences of LPPs from different organisms showed that *S. coelicolor* LPPs (scoLPPs) are evolutionary associated with synLPP, in the subgroup of prokaryotic LPPs (Figure 2). Non-plastidic LPPs of *A. thaliana* (atLPPα1, 2, 3) are clustered together with LPPs from human, murine and *S. cerevisiae* in a clade merely composed by eukaryotes LPPs (Figure 2). In agreement with Nakamura *et al.*[37], our studies also show that bacterial LPPs group closer to the clade of “plastidic LPPs with prokaryote origin” (atLPPδ, ε1 and ε2) than to the eukaryotic one.

Additional bioinformatic analysis of these sequences revealed that scoLppα (Sco1102) orthologues are conserved in all the available *Streptomyces* genomes, as well as in other species of actinomycetes belonging to the genus *Mycobacterium*, *Rhodococcus* and *Nocardia,* among others. The presence of scoLppβ (Sco1753) homologous sequences is however limited to the *Streptomyces* genus, being present in all the species sequenced up to date. Finally, scoLppγ (Sco6355) coding sequence is conserved only in *S. coelicolor* and in *S. avermitilis*. This gene is part of a five member operon (SCO6353-SCO6357) that also encodes for a putative sensor histidine kinase and a response regulator. The genome organization of scoLppγ and the fact that this operon is absent in other actinomycetes, suggest a specific role of this protein in *S. coelicolor* and *S. avermitilis* metabolism.

Overall, orthologues sequences to scoLppα and scoLppβ are widely distributed among oleaginous bacteria, merely actinomycetes; thus these two proteins can be considered as the best PAP candidates.

### Heterologous expression of *S. coelicolor* Lppα, Lppβ and Lppγ in *E. coli*

**Figure 3:**
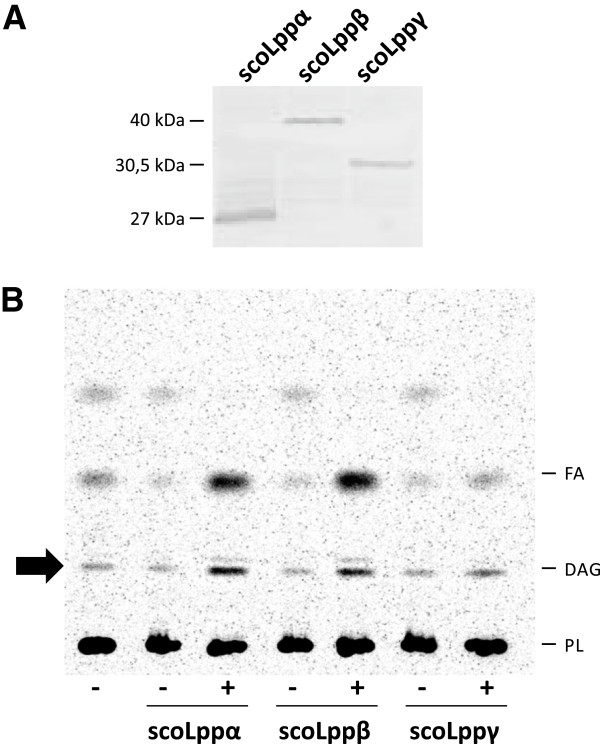
**Heterologous expression of scoLPPs in *E. coli *and analysis of their lipid profile.** (**A**) Immunoblot analysis of *E. coli* soluble fractions with anti-His antibodies. (**B**) Total lipid extracts from [^14^C] acetic acid-labelled cultures of the indicated *E. coli* strains were analyzed on silica gel TLC plates and developed in hexane:diethylether:acetic acid (70:30:1, v/v/v). FA: Fatty Acids. DAG: Diacylglycerol. PL: Phospholipid. (+): Inducer added. (−): No inducer.

To initiate the functional characterization of Lppα, Lppβ and Lppγ, an N-terminal His-tag version of each gene was cloned under the control of *P*_*BAD*_ promoter in the pBAD33 vector [43]. Plasmids pBAD-LPPα, pBADL-LPPβ and pBAD-LPPγ were introduced by transformation in *E. coli* C41 (DE) strain, which is suitable for over-production of membrane proteins [44]. Transformed cells were grown to mid-log phase and then cultivated for 16 h at 23°C after induction with L-arabinose. Immunoblot analysis of the corresponding *E. coli* soluble homogenates showed that each His-tagged protein migrated according to the molecular weight predicted for the polypeptides His-Lppα (27 KDa), His-Lppβ (40 KDa) and His-Lppγ (30,5 kDa), respectively (Figure 3A). Then, we analyzed the total lipid profile of these recombinant strains by metabolic labeling with [^14^C]-acetate. We found out that Lppα and Lppβ were able to raise the intracellular levels of DAG 3 to 6-fold higher than that of the parental strain (Figure 3B). However, no modification in total lipid pattern, and particularly in DAG levels, was observed in the strain containing Lppγ, suggesting either that this protein is not a functional PAP in this background or that it catalyzes a different reaction. The fatty acid content of cells expressing Lppα and Lppβ was also increased when compared to the non-induced strains (Figure 3B). This effect could be due to an increased FA biosynthesis or to a higher recycling-degradation of labeled lipids as a consequence of increasing DAG levels.

To evaluate Mg^2+^-independent PAP activity of the *Streptomyces* proteins, we used purified *E. coli* membrane homogenates of C41 (DE) strains expressing each of the three genes under study. As shown in Table 1, membrane proteins isolated from the strain expressing Lppα and Lppβ displayed a considerable increase in PAP activity compared with the control strain (C41 (DE) transformed with pBAD33). The highest difference was observed for Lppβ, with an increase of 5-fold (Table 1). Cells expressing Lppγ did not present difference in PAP activity relative to the control (Table 1).

**Table 1:** **Phosphatidic acid phosphatase activity in membranes of *E. coli *expressing scoLPPs**

**Strain**	**Specific PAP activity**^**a**^
C41(DE3)/pBAD33	0.9 ± 0.2
C41(DE3)/pBAD-LPPα	2.51 ± 0.06
C41(DE3)/pBAD-LPPβ	4.5 ± 0.1
C41(DE3)/pBAD-LPPγ	1.02 ± 0.08

^a^Values represent the mean S.D. of triplicate determinations.

### Lppα and Lppβ complement the temperature sensitive phenotype of *dpp1lpp1pah1-*deficient yeast cells

**Figure 4:**
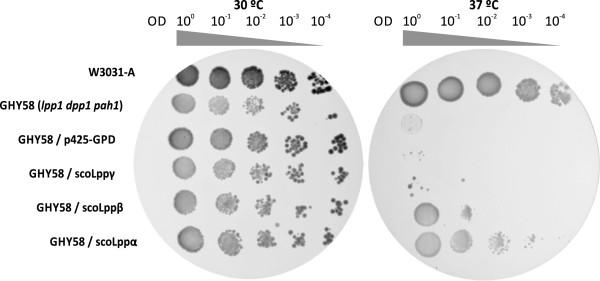
**Complementation of the temperature sensitive phenotype of *dpp1 lpp1 pah1 *yeast knockout strain by expressing scoLPPs.** W3031A: wild type strain; GHY58 (*lpp1dpp1pah1*): triple *knockout* mutant strain; GHY58/p425GPD, GHY58/scoLppγ, GHY58/scoLppβ, GHY58/scoLppα: triple *knockout* strain transformed with p425GPD empty vector and with p425GPD expressing scoLppγ, scoLppβ or scoLppα. 10 μl of each dilution were spotted on the plates, and followed by incubation at 30 and 37°C for two days.

To continue with the functional characterization of Lppα, Lppβ and Lppγ, each coding gene was linked to the constitutive GPD promoter in the yeast expression vector p425GPD [45]. The resulting constructs were transformed into *S. cerevisiae* GHY58 (*dpp1lpp1pah1*) mutant strain, which displays several phenotypes such as severe growth deficiency at 37°C, reduced levels of PAP activity, elevated levels of PA and decreased levels of DAG and TAG [25]. Strains GHY58, GHY58*/*p425-LPPα, GHY58*/*p425-LPPβ, GHY58*/*p425-LPPγ, GHY58*/*p425GPD (control) and WH303-1A (wild type) were cultivated in YPD media during 48 h at 30°C, normalized to OD_600nm_ 1 and serially diluted. The dilutions were plated on YPD media and then incubated at 37°C and 30°C. We observed that strains GHY58*/*p425LPPα and GHY58*/*p425LPPβ incubated at 37°C displayed growth on dilutions as low as 1.0×10^-3^ and 1.0×10^-1^, respectively (Figure 4); while GHY58*/*p425GPD and cells expressing Lppγ (GHY58*/*p425-LPPγ) only grew, although poorly, at OD_600_ 1 at 37°C. At 30°C all cells lines presented comparable growth, indicating that complementation of temperature-sensitive phenotype resulted from Lppα and Lppβ expression. As a control for plasmid maintenance, all the strains were plated on selective conditions (synthetic complete medium plus glucose 2% and omitting the corresponding amino acids). Furthermore, [^14^C]-acetate labeling assays indicated that the expression of Lppα and Lppβ in this mutant background increased the pools of DAG 3.2-fold and 2.2-fold respectively, relative to the wild type strain; while GHY58*/*p425-LPPα and GHY58*/*p425-LPPβ did not restore the wild type levels of TAG (data not shown).

Since the heterologous expression of Lppγ did not show a clear effect in lipid metabolism, neither in *E. coli* nor in *S. cerevisiae*, we continued the characterization of the other two *S. coelicolor* PAP candidates.

### Lppα and Lppβ are involved in TAG biosynthesis in *S. coelicolor*

**Figure 5:**
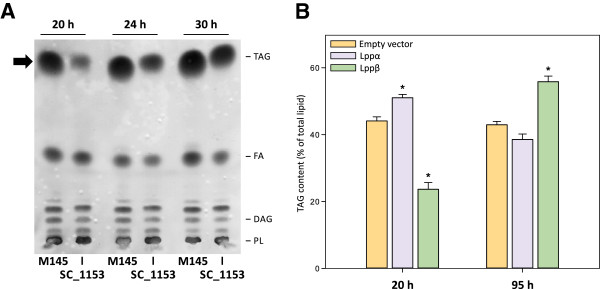
**Analysis of the *in vivo *role of Lppα and Lppβ in TAG accumulation.** (**A**) Total lipid extracts from cultures of the indicated *S. coelicolor* strains grown on SMM medium during 20, 24 and 30 h, were analyzed on silica gel TLC plates and developed in hexane:diethylether:acetic acid (70:30:1, v/v/v). (**B**) Quantification of TAG content of *S. coelicolor* strains overexpressing the indicated scoLPPs. Stars mean significant differences respect to empty plasmid control strain (p < 0.05). TAG: Triacylglycerol. FA: Fatty Acid. DAG: Diacylglycerol. PL: Phospholipid.

The *in vivo* role of Lppα and Lppβ in storage lipid synthesis was studied by generating single mutants in each of the genes encoding for the putative PAPs and also a double mutant strain. For this, each open reading frame was disrupted with a Tn5-derivative transposon (see Materials and Methods for details). TAG formation in the three mutant strains was analyzed by total lipid extraction and fractionation by normal-phase TLC. In all conditions tested, the single mutants did not show a significant effect on TAG accumulation (data not shown). However, in nitrogen-starving and carbon excess conditions (SMM media), where TAG synthesis is strongly induced, the *lppα lppβ* double mutant strain (SC_1153) presented a significant reduction in TAG content at exponential growth phase (Figure 5A). A densitometric quantification of each lipid type indicated that DAG content is about 30% lower than that of the wild type strain, while TAG content showed a reduction from 65% to 40% throughout growth compared with the M145 strain. Further analysis revealed a decrease of 28.2% in membrane-associated PAP activity of SC_1153 strain (3.1 ± 0.2 U/mg), respect to the wild type strain (4.29 ± 0.09 U/mg). The absence of phenotype in the single mutants suggests the existence of a compensating effect that can mitigate the disruption of the individual PAP enzymes. In this sense, expression of either Lppα or Lppβ in a SC_1153 genetic background was sufficient to restore TAG accumulation to wild type levels (data not shown). These results indicate that both genes products contribute to TAG biosynthesis in *S. coelicolor* and are necessary to achieve wild type levels of this storage compound, at least under the growth conditions tested. Moreover, the fact that the double mutant strain still produces considerable amounts of TAG also indicate that the remaining PAP activity present, as well as alternative pathway(s), can also generate the DAG needed for TAG biosynthesis in this strain.

To further evaluate the *in vivo* role of Lppα and Lppβ in TAG biosynthesis, we constructed M145 derivative strains, each containing an extra copy of the Lppα or the Lppβ encoding genes under the transcriptional control of *P*_ermE*_[46]. The strains were named SC_Lppα, SC_Lppβ and SC_285 (control strain carrying the empty vector; Table 2). The recombinant strains were grown in different media and their ability to synthesize TAG was determined by total lipid extraction, TLC fractionation and densitometry quantification. Both SC_Lppα and SC_Lppβ strains reached lower cell density in liquid cultures of minimal SMM medium compared with the wild type strain, whereas timing of transition and stationary phase entrance was not affected (data no shown). This is not an unexpected observation, since overexpression of a PAP enzyme would shift the flux of PA to TAG, in detriment of glycerophospholipids synthesis; alternatively, by means of this reaction, DAG can be accumulated causing toxicity to the cell [47]. A third cause could be the merely effect of overexpressing a membrane protein. Therefore, although SMM medium is routinely used to analyze storage lipid content we had to conduct all these experiments in R5 rich media, where growth was less affected. As shown in Figure 5B, overexpression of LPPα caused an increase of 15.7% in the accumulation of TAG compared with the control strain at the early stationary phase of growth. Interestingly, lipid profile analysis of SC_Lppβ overexpressing strain revealed an increase in the content of this neutral lipid, up to 29.9% compared with control strain, but in the late-stationary phase of growth (Figure 5B). Further, this effect was accompanied by a higher *de novo* TAG synthesis as determined by [^14^C]-acetate labeling assays (data not shown).

**Table 2:** Strains and plasmids

**Strain or plasmid**	**Description**	**Reference**
**Strains**		
***S. coelicolor***		
M145	Parental strain, SCP1^-^ SCP2^-^	[46]
SC_1102	SCO1102::*Tn5062*, derivative of M145; Am^R^	This study
SC_1753	SCO1753::*Tn5062*, derivative of M145; Am^R^	This study
SC_1153	SCO1102::*Tn5062* SCO1753::*Tn5066*, derivative of SC_1102; Am^R^ Hyg^R^	This study
SC_285	M145 *attB*_*ΦBT1*_::pTR285; Km^R^	This study
SC_Lppα	M145 *attB*_*ΦBT1*_::p285-LPPα; Km^R^	This study
SC_Lppβ	M145 *attB*_*ΦBT*1_::p285-LPPβ; Km^R^	This study
SC_P1102	SC_1153 *attB*_*ΦBT1*_::pRT802-LPPα; Am^R^ Hyg^R^ Km^R^	This study
SC_P1753	SC_1153 *attB*_*ΦBT*1_::pRT802-LPPβ; Am^R^ Hyg^R^ Km^R^	This study
***E. coli***		
DH5α	*E. coli K12 F*^*-*^*lacU169 (Φ80lacZΔM15) endA1 recA1 hsdR17 deoR supE44 thi-1 l2 gyrA96 relA1*	[48]
BL21 (DE3)	*F*^*–*^*ompT gal dcm lon hsdS*_*B*_*(r*_*B*_^*-*^*m*_*B*_^*-*^*) λ(DE3)*	Novagen
C41 (DE3)	*F*^*–*^*ompT gal dcm lon hsdS*_*B*_*(r*_*B*_^*-*^*m*_*B*_^*-*^*) λ(DE3)*	Lucigen
ET 12567	*supE44 hsdS20 ara*^*-*^*14 proA2 lacY galK2 rpsL20 xyl*^*-*^*5 mtl*^*-*^*1 dam*^*-*^*dcm*^*-*^*hsdM*^*-*^; Cm^R^	[49]
***S. cerevisiae***		
W303-1A	*MAT*a *ade2-1 can1-100 his3-11,15 leu2-3,112 trp1-1 ura3-1*	[50]
GHY58	*W303-1A dpp1*_::*TRP1*/*Kan*^*R*^*lpp1*_::*HIS3/Kan*^*R*^*pah1*_::*URA3*	[25]
**Plasmids**		
pET28a	Vector for expression of N terminal His-tagged proteins under the strong T7 promoter; Km^R^	Novagen
pBAD33	Vector for recombinant protein expression under the control of the *P*_*BAD*_ promoter; Cm^R^	[43]
pCR®-BluntII-TOPO	Vector used for cloning of blunt PCR products; Km^R^	Invitrogen
pUZ8002	RK2 derivative with defective *oriT*; Km^R^	[49]
pKOS111-47	RK2 derivative with defective *oriT*; Ap^R^	B. Julien (Personal communication)
pQM5066	Plasmid carrying a copy of *Tn5066*; Hyg^R^	P. Dyson, (Personal communication)
pRT802	Intregrative vector based on ΦBT1 phage integrase; Km^R^	[51]
p425GPD	Multicopy Yeast/*E. coli* expression vector with GPD promoter and LEU2 marker; Ap^R^	[45]
pTR285	pRT802 derivative carrying the *P*_ermE*_ promoter with no gene under its control; Km^R^	[52]
pBAD0958	pBAD33 carrying the SCO0958^His^ gene under the control of *P*_*BAD*_ promoter; Cm^R^	[52]
pBAD-LPPα	pBAD33 carrying the SCO1102^His^ gene under the control of *P*_*BAD*_ promoter; Cm^R^	This study
pBAD-LPPβ	pBAD33 carrying the SCO1753^His^ gene under the control of *P*_*BAD*_ promoter; Cm^R^	This study
pBAD-LPPγ	pBAD33 carrying the SCO6355^His^ gene under the control of *P*_*BAD*_ promoter; Cm^R^	This study
p425-LPPα	p425-GPD carrying the SCO1102^His^ gene under the control of GPD promoter; Ap^R^, LEU2	This study
p425-LPPβ	p425-GPD carrying the SCO1753^His^ gene under the control of GPD promoter; Ap^R^, LEU2	This study
p425-LPPγ	p425-GPD carrying the SCO6355^His^ gene under the control of GPD promoter; Ap^R^, LEU2	This study
p28-LPPα	pET28 carrying the SCO1102^His^ gene under the control of T7 promoter; Km^R^	This study
p28-LPPβ	pET28 carrying the SCO1753^His^ gene under the control of T7 promoter; Km^R^	This study
p285-LPPα	pTR285 carrying the SCO1102^His^ gene under the control of *P*_ermE*_ promoter; Km^R^	This study
p285-LPPβ	pTR285 carrying the SCO1753^His^ gene under the control of *P*_ermE*_ promoter; Km^R^	This study
pRT802-LPPα	pRT802 carrying the SCO1102^His^ gene under the control of its own promoter; Km^R^	This study
pRT802-LPPβ	pRT802 carrying the SCO1753^His^ gene under the control of its own promoter; Km^R^	This study

These experiments are in agreement with the *in vivo* and *in vitro* studies mentioned before, and indicate that both Lppα and Lppβ are functional PAPs involved in TAG biosynthesis in *S. coelicolor*.

### Co-expression of *S. coelicolor* PAP and DGAT enzymes leads to TAG biosynthesis in *E. coli*

**Figure 6:**
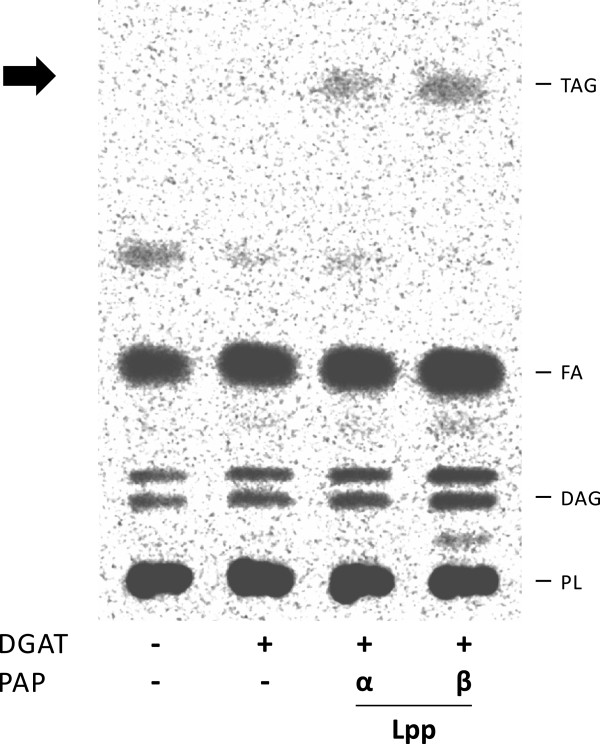
**Reconstitution of *S. coelicolor de novo *TAG biosynthesis pathway in *E. coli*.** Total lipid extracts from [^14^C] acetic acid-labeled cultures of the indicated *E. coli* strains were analyzed on silica gel TLC plates and developed in hexane:diethylether:acetic acid (70:30:1, v/v/v). TAG: Triacylglycerol. FA: Fatty Acid. DAG: Diacylglycerol. PL: Phospholipid.

In a previous work we reported that heterologuos expression of the DGAT (diacylglycerol:acyltransferase) Sco0958 from *S. coelicolor* in an *E. coli dgk* mutant, leads to the accumulation of TAG in this host [52]. We employed a *dgk* (diacylglycerol kinase) mutant strain because it presents higher levels of DAG than the wild type strain [52]; thus, this lipid could be used as substrate by the DGAT for TAG biosynthesis. In this sense, the single expression of Sco0958 in wild type *E. coli* did not generate detectable amounts of TAG [52]. To analyze whether we could reconstitute the complete *S. coelicolor* TAG biosynthesis pathway in *E. coli*, we co-expressed each of the identified PAPs Lppα or Lppβ and the DGAT Sco0958. For this, we constructed a BL21 (DE3) derivative strain, where Sco0958 is expressed under the control of *P*_*BAD*_ (pBAD0958, Table 2). The strain BL21/pBAD0958 was transformed with plasmids p28-LPPα or p28-LPPβ, giving the strains BL21/pBAD0958/p28-LPPα and BL21/pBAD0958/p28-LPPβ, respectively. Each recombinant strain was grown until mid-log phase and treated as described in Material and Methods. We found out that co-expression of either Lppα or Lppβ together with Sco0958 DGAT enzyme, led to TAG production in a wild type *E. coli* strain (Figure 6). These results clearly demonstrate that these enzymes are sufficient to synthesize *de novo* TAG using precursors from the glycerophospholipid metabolism of a non-oleaginous host.

## Conclusions

In this work we described the identification and physiological characterization of the first phosphatidic phosphatase enzymes involved in triacylglycerol biosynthesis in bacteria.

By means of a comprehensive bioinformatic analysis of the *S. coelicolor* genome, we were able to identify Lppα and Lppβ as the best PAP candidates of this microorganism. They were both predicted to be integral membrane proteins belonging to the PAP2 family of enzymes also known as LPPs (pfam01569; Figure 2). Heterologous expression of *S. coelicolor* PAPs (scoLPPs) in *E. coli* increased the intracellular levels of DAG up to 6-fold and enhanced PAP activity in their membrane fractions up to 5-fold when compared with the control strain (Figure 3B, Table 1). Furthermore, when expressed in yeast, scoLPPs were able to complement the temperature sensitive phenotype of a *dpp1 lpp1 pah1* deficient mutant (Figure 4), and to raise the intracellular concentration of DAG.

To unravel the physiological role of Lppα and Lppβ in *S. coelicolor*, we constructed the corresponding simple and double mutant strains. Disruption of either *lppα* or *lppβ* caused no alteration in the intracellular levels of TAG. However, inactivation of both genes, *lppα* and *lppβ,* led to a clear reduction of the membrane associated PAP activity, as well as a reduction of TAG accumulation (Figure 5A). Further, overexpression of these genes in a wild type background provoked an increase of 15.7% and 29.9% in the content of TAG for Lppα and Lppβ, respectively (Figure 5B). These observations support an active role of these two PAP enzymes in the TAG biosynthesis pathway of *S. coelicolor*.

Finally, co-expression of the *S. coelicolor* DGAT enzyme Sco0958 together with Lppα or Lppβ in *E. coli* reconstituted the complete pathway for *de novo* TAG biosynthesis in a heterologous host (Figure 6).

The identification of Lppα and Lppβ completes the minimal set of enzymes, together with the three DGATs previously characterized, required for *de novo* TAG biosynthesis in *S. coelicolor*. Remarkably, the overexpression of these PAPs in *Streptomyces* bacteria contributes to a higher productivity of this single cell oil.

Overall, these results provide new elements and tools for future cell engineering towards achieving sustainable and cost-effective single cell oil production in oleaginous bacteria. Furthermore, the successful reconstruction of the specific TAG biosynthesis pathway in a heterologous host with good fermentation properties such as *E. coli* broadens the bacterial platforms for next-generation biofuels production.

## Methods

### Strains, media and growth conditions

The strains and plasmids used in this study are described in Table 2*. E. coli* strains were grown either on solid or in liquid Luria-Bertani (LB) medium at 37°C and supplemented when needed with the following antibiotics: 100 μg ml^-1^ ampicillin (Ap), 50 μg ml^-1^ kanamycin (Km), 20 μg ml^-1^ chloramphenicol (Cm), 100 μg ml^-1^ hygromycin (Hyg) or 50 μg ml^-1^ apramycin (Am). Yeast cells were grown in YPD medium (1% yeast extract, 2% peptone, 2% glucose) or in synthetic complete (SC) medium containing 2% glucose at 30°C [53]. For selection of yeast cells bearing plasmids, appropriate amino acids were omitted from SC medium. *Streptomyces* strains were grown at 30°C in liquid media R5 or SMM and on solid media MS, R5, or in SMMS containing 1% w/v glucose (SMMS-glucose) [46]. The antibiotics Am, Hyg and Km were added at final concentrations of 50 μg ml^-1^ to solid medium respectively.

### Construction of *lppα* and *lppβ* mutants and the *lppα lppβ* double mutant strain of *S. coelicolor*

Disruption of the open reading frames SCO1102 (*lppα*) and SCO1753 (*lppβ*) was carried out by homologous recombination using cosmids from the transposon mutant ordered cosmid library of *S. coelicolor*[54]. Cosmids 6A05.2.b04 and I34.1.D02, carrying an individual Tn5062 insertion in SCO1102 and SCO1753 genes, respectively, were introduced into *S. coelicolor* M145 by conjugation using *E. coli* ET12567/pUZ8002 as donor. Three independent Am^R^ Km^S^ exconjugants were isolated from each conjugation and checked by PCR, verifying that allelic replacement had occurred. The SCO1102 disruption was analyzed with 1102_F/R, 1102_F/EZL2, and 1102_R/EZR1 primer pairs; and for the inactivation of SCO1753 oligonucleotides 1753_F/R, 1753_F/EZR1 and 1753_R/EZL2 were used (Table 3).

**Table 3:** Primers

**Name**	**Sequence (5’-3’)**	**Reference**
EZR1	ATGCGCTCCATCAAGAAGAG	[54]
EZL2	TCCAGCTCGACCAGGATG	[54]
SCO1102_F	CCATATGCAGACGCCGCCGGTCGAC	This study
SCO1102_R	TGGCCGAATTCTAGGGGGCGAAGGA	This study
SCO1753_F	CGATCATATGCGTACCGAACGGAAG	This study
SCO1753_R	CCCCGAATTCACCCGAACGACACC	This study
SCO6355_F	GGGTCATATGAAGCGCGGCGATGTC	This study
SCO6355_R	GTGAATTCCGGGGACGGTGTCGAAG	This study
P1102_F	GCGGCCGCCGACGGTGCCTTGTGG	This study
P1102_R	ACTAGTGGGGGCGAAGGATGCGACC	This study
P1753_F	GCGGCCGCGGGGTCGCGCTCCTGGT	This study
P1753_R	ACTAGTACCCGAACGACACCCCCTG	This study

Restriction sites are shown underlined.

In order to generate the double mutant strain, the Am resistance marker of cosmid I34.1.D02 was replaced by the Hyg resistance cassette of pQM5066 (P. Dyson, personal communication). Conjugal transfer of cosmid I34.1.D02 Hyg^R^ to SC_1102 yielded Am^R^ Hyg^R^ Km^S^ exconjugants. Three independent clones were picked and checked by PCR with the primer pairs described above for the mutations in SCO1102 and SCO1753.

### Cloning of *lppα, lppβ* and *lppγ* genes

*lppα* (SCO1102), *lppβ* (SCO1753) and *lppγ* (SCO6355) were amplified from *S. coelicolor* M145 genomic DNA by PCR with primers SCO1102_F/R, SCO1753_F/R and SCO6355_F/R, respectively. The resulting PCR products were cloned in pCR®-BluntII-TOPO vector and checked by DNA sequencing. The DNA fragments containing *lppα*, *lppβ* and *lppγ* genes were cloned as NdeI/EcoRI digests in pET28a, to yield plasmids p28-LPPα, p28-LPPβ and p28-LPPγ. pET28 derivative plasmids containing each of the putative PAP sequences were digested with XbaI/HindIII and the restriction fragments were cloned in the same sites of pBAD33, yielding pBAD-LPPα, pBAD-LPPβ, pBAD-LPPγ. In order to express PAP candidate genes in *S. cerevisiae*, the XbaI/HindIII digestion fragments from the pET28 derivatives (p28-LPPα, p28-LPPβ, p28-LPPγ) were cloned in the SpeI/HindIII sites of p425GPD vector, yielding p425-LPPα, p425-LPPβ and p425-LPPγ. To achieve overexpression of *lppα* and *lppβ* genes in *S. coelicolor*, the pET28 derivative plasmids containing these genes were digested with XbaI/HindIII and the fragments were cloned in the SpeI/HindIII sites of pTR285 vector, yielding p285-LPPα and p285-LPPβ.

To complement the *S. coelicolor* double mutant strain SC_1153, we amplified SCO1102 and SCO1753 genes plus 300 or 500 bp upstream sequences (in order to include the native promoter) with primers P1102_F/R and P1753_F/R, respectively. The resulting PCR products were cloned in pCR®-BluntII-TOPO vector and checked by DNA sequencing. These sequences were cloned as NotI/SpeI digestion fragments in pRT802 vector, yielding plasmids pRT802-LPPα and pRT802-LPPβ.

All the plasmids described in this section are listed in Table 2.

### Membrane preparation of *E. coli* and *S. coelicolor* strains

*E. coli* C41 strains harbouring plasmid pBAD-LPPα, pBAD-LPPβ and pBAD-LPPγ were grown in LB at 37°C until OD_600nm_ 0.6. Then, protein expression was induced by addition of L-arabinose 0.2% and it was followed by overnight incubation at 23°C with gently shaking. Cells were harvested by centrifugation at 4,000 × g for 20 min at 4°C, washed twice with Buffer A (Tris–HCl pH 7.5 50 mM, NaCl 100 mM, EDTA 1 mM, β-mercaptothanol 10 mM) and resuspended in the same buffer. The next steps were all done at 4°C. Disruption of the cells was carried out by sonication (Vibra-Cell™, Sonics & Material Inc.), in the presence of 1 mM phenylmethylsulfonyl fluoride (PMSF) and avoiding foaming. The lysate was cleared by centrifugation at 15,000 × g for 30 min to separate cell debris, and the supernatant was ultracentrifuged at 120,000 × g for 2 h to pellet the membrane fraction. The resulting pellet was washed twice with buffer B (Tris–HCl pH 7.5 20 mM, β-mercaptothanol 10 mM, PMSF 0.5 mM), and resuspended in the same buffer. Protein concentration was quantified by Lowry assay using BSA as standard [55].

*S. coelicolor* strains were grown in the corresponding media indicated for each experiment. Cells were harvested by centrifugation at 4,000 × g for 20 min at 4°C washed twice with Buffer A and resuspended in the same buffer. The next steps were done as described above for *E. coli*.

### SDS-PAGE and immunoblot

SDS-PAGE and immunoblot analysis using nitrocellulose membranes were carried out using standard protocols [53,56]. For detection of the His-tagged proteins, mouse monoclonal anti-His antibodies (QIAGEN™) were used at a dilution of 1:1,000. Anti-mouse IgG-alkaline phosphatase conjugates were used as secondary antibodies at a dilution of 1:3,000. His-tagged proteins were visualized by immunoblots using chromogenic detection as described by the manufacturer.

### Phosphatidic acid phosphatase activity assay

To test the phosphatase activity of the putative PAPs of *S. coelicolor* we used phosphatidic acid as the enzyme substrate. The diacylglycerol generated on the reaction was measured by LC-MS/MS. Standard phosphatase assays were performed in a 100 μL reaction mixture containing 25 mM Tris–HCl, pH 7.5, 2.5 mM Triton X-100 detergent and dipalmitoylphosphatidic acid (DPPA; Avanti Polar Lipids, Alabama, USA) 0.25 mM as substrate. Aliquots of membrane fractions of the corresponding strain were added to initiate the reaction, and after incubation at 30°C the reactions were quenched by adding methanol:chloroform (2:1). Subsequent lipid extraction was performed by the addition of chloroform and distilled water. The organic phase was dried, solubilised in 50 μL of mobile phase and 5 μL aliquots were injected for HPLC and LC-MS/MS analysis. The organic extracts were separated on a ZORBAX Eclipse XDB-C8 column (3.0 × 50 mm, particle size = 1.8 μm; Agilent, USA) using a binary solvent system of water (Solvent A) and methanol (Solvent B). A linear gradient from 80% B to 100% B was applied between 0 and 25 minutes. Both solvents were supplemented with 5 mM ammonium acetate. The outlet of the liquid chromatograph was connected to a micrOTOF mass spectrometer (Bruker Daltonik, Bremen, Germany) operating in the positive-ion mode. The data was acquired online in the mass range m/z 35–1000. Dipalmitoylglycerol (DPG) was detected as a the transition [M + NH_4_]^+^ → [M-R-OH]^+^ ion (m/z 586.5 → m/z 313.3). A calibration curve was done using DPG as standard (Avanti Polar Lipids, Alabama, USA), in the same conditions as the phosphatase reaction. DAG concentration in the samples was calculated by the linear regression equation obtained from the calibration curve.

A unit of enzymatic activity was defined as the amount of enzyme that catalyzed the formation of 1 nmol of product/min. Specific activity was defined as units/mg of protein. PAP activity was linear-dependent to time and protein concentration within the range tested.

### Total lipid analysis

*E. coli* strains harboring pBAD-LPPα, pBAD-LPPβ and pBAD-LPPγ were grown in LB media at 37°C until OD_600nm_ 0.6. Then, protein expression was induced by addition of L-arabinose 0.2%, 3 μCi [^14^C]-acetate was added at the same time and the culture was kept overnight at 23°C. For Sco0958-PAPs co-expression experiment, cells at OD_600nm_ 0.6 were induced by L-arabinose 0.2% and IPTG 0.1 mM. 3 μCi [^14^C]-acetate was added to the culture, and it was kept overnight at 23°C. Total lipids of *E. coli* strains were extracted as described by Bligh & Dyer [57] directly from ^14^C labeled cells.

For *S. coelicolor* strains, total lipids were extracted twice from lyophilized cell material (2 mg) with chloroform/methanol (2:1 v/v) as previously described [58]. For *de novo* lipid biosynthesis, *S. coelicolor* was grown in R5 liquid media and 3 ml of culture was labeled for 3 h with 3 μCi [^14^C]-acetate (58.9 mCi/mmol, PerkinElmer).

The lipid extracts were dried and analyzed by TLC on silica gel 60 F254 plates (0 ± 2 mm, Merck), using the solvent systems hexane/diethylether/acetic acid (70:30:1, v/v/v) [59]. Lipid fractions were visualized by Cu-phosphoric staining and identified by comparing to the mobility of known standards. For ^14^C labeled lipids the radioactivity incorporated into each lipid fraction was quantified using a Storm 860 PhosphorImager (Molecular Dynamics) and the corresponding spots were quantified using ImageQuant software (version 5.2).

## Competing interests

The authors declare that they have no competing interests.

## Authors’ contributions

SC, SMB, AA and HG designed all the experiments. SC did the sequences bioinformatic analysis and constructed the plasmid backbones. SC, SMB and AA performed all the experiments. AA and HG wrote the manuscript. All the authors read and approved the final manuscript.
